# LittleFaceNet: A Small-Sized Face Recognition Method Based on RetinaFace and AdaFace

**DOI:** 10.3390/jimaging11010024

**Published:** 2025-01-13

**Authors:** Zhengwei Ren, Xinyu Liu, Jing Xu, Yongsheng Zhang, Ming Fang

**Affiliations:** 1 School of Artificial Intelligence, Changchun University of Science and Technology, Changchun 130012, China; renzhengwei@cust.edu.cn (Z.R.); xujing@cust.edu.cn (J.X.); zys@cust.edu.cn (Y.Z.); fangming@cust.edu.cn (M.F.); 2 Zhongshan Institute of Changchun University of Science and Technology, Zhongshan 528400, China

**Keywords:** face recognition, RetinaFace, Adaface, object tracking, deep learning, low resolution

## Abstract

For surveillance video management in university laboratories, issues such as occlusion and low-resolution face capture often arise. Traditional face recognition algorithms are typically static and rely heavily on clear images, resulting in inaccurate recognition for low-resolution, small-sized faces. To address the challenges of occlusion and low-resolution person identification, this paper proposes a new face recognition framework by reconstructing Retinaface-Resnet and combining it with Quality-Adaptive Margin (adaface). Currently, although there are many target detection algorithms, they all require a large amount of data for training. However, datasets for low-resolution face detection are scarce, leading to poor detection performance of the models. This paper aims to solve Retinaface’s weak face recognition capability in low-resolution scenarios and its potential inaccuracies in face bounding box localization when faces are at extreme angles or partially occluded. To this end, Spatial Depth-wise Separable Convolutions are introduced. Retinaface-Resnet is designed for face detection and localization, while adaface is employed to address low-resolution face recognition by using feature norm approximation to estimate image quality and applying an adaptive margin function. Additionally, a multi-object tracking algorithm is used to solve the problem of moving occlusion. Experimental results demonstrate significant improvements, achieving an accuracy of 96.12% on the WiderFace dataset and a recognition accuracy of 84.36% in practical laboratory applications.

## 1. Introduction

Face recognition is a visual processing technology that automatically identifies individuals based on their facial features. In recent years, face recognition technology has been widely applied in fields such as face-based payments, attendance systems, and identity verification [1]. Compared with traditional biometric technologies, face recognition technology is characterized by multi-feature capabilities, concealment, non-contact operation, and low cost, enabling technologies originally used in stringent identity verification contexts to proliferate on mobile devices [2]. However, current face recognition systems are primarily static, requiring the person to be recognized to remain stationary in front of the capturing camera during the recognition process. The device then matches the detected face with faces in a database, a method that necessitates cooperation from the individual being recognized. In contrast, dynamic face recognition does not require cooperation from the person being recognized and can identify identities in unrestricted, unconstrained conditions [3]. In unconstrained scenarios, factors such as changes in the recognized person’s facial expressions, posture, and lighting intensity [4], the proportion of occlusion, and varying sizes of captured facial images [5] interfere with the overall face recognition process. In real-life scenarios, these factors randomly and collectively affect face recognition, making it challenging to accurately determine the identity of the person being recognized in actual dynamic settings. Deep learning is widely applied in the field of face recognition [6]. It can extract deep-level features, and training using vast datasets enhances the model’s robustness and broader adaptability, resulting in excellent performance in detection and recognition effects. Recently, face recognition has made significant progress by referencing general advancements in object detection and deep learning. CNN-based object detectors can be divided into two-stage detection algorithms and one-stage detection algorithms based on regression [7]. Recent cutting-edge face recognition methods focus on adopting dense sampling within a single-stage framework, which demonstrates good performance in both speed and accuracy compared to two-stage methods [8].

With the advancement of technology, low-quality images are increasingly becoming an important component of face recognition datasets, as they are encountered in surveillance videos and drone footage. Given that state-of-the-art face recognition (FR) methods [9,10] can achieve over 98% verification accuracy on relatively high-quality datasets such as LFW or CFP-FP [11,12], recent FR challenges have shifted to lower-quality datasets like IJB-B, IJB-C, and IJB-S [13,14]. Despite the challenge of achieving high accuracy on low-quality datasets, most popular training datasets still consist of high-quality images [9,15,16], with only a small portion of training data being of low quality. One issue with low-quality face images is that they are often unrecognizable. When the image degradation is too significant, relevant identity information disappears from the image, resulting in unrecognizable images. These unrecognizable images are detrimental to the training process because the model will attempt to utilize other visual features, such as clothing color or image resolution, to reduce the training loss. If these images dominate the distribution of low-quality images, the model’s performance on low-quality datasets during testing may be poor.

For such low-resolution images, two primary approaches have been explored to address this issue: (1) construction-based and (2) projection-based methods. Construction-based methods involve enhancing the visual quality of the low-resolution (LR) input prior to recognition, known as face super-resolution (FSR). In this way, the FR process is divided into two tasks: identity-preserving FSR and super-resolution face recognition (SRFR). Special attention has been given to Generative Adversarial Networks (GANs) [16,17,18] within the face generation module. Although GANs achieve remarkable outputs in terms of image quality and human perception, they add high-frequency components to the synthesized images, which adversely affect the recognition process [19]. Furthermore, FSR is an ill-posed problem due to the existence of multiple high-resolution (HR) faces for each LR image [19]. Additionally, face images are influenced by several other covariate factors (esthetics), such as head pose, lighting, and expression. These factors result in a significant gap between the feature embeddings of HR and SR faces in the identity metric space, which significantly degrades the final FR performance.

Projection-based methods aim to create a shared embedding space that can accommodate both HR and LR images. To achieve this, synthetic LR data can be utilized to enhance the resolution diversity of the dataset [20,21]. However, due to the fixed angular margin in traditional FR methods, there are convergence issues, and they do not adapt well to data augmentations such as downsampling or random cropping [22,23]. To address this problem, methods based on adaptive margin adjustment according to sample difficulty have been proposed [23,24]. MagFace suggests using feature norms as a metric of image quality and adjusting the margin accordingly. Adaptive margins have solved the convergence problem to some extent. However, performance still deteriorates significantly when dealing with LR images [25]. For instance, the face verification accuracy on LFW is typically above 99%. Yet, the performance of Tinyface is around 59%. Furthermore, Nourelahi et al. [26] demonstrated that training models on perturbed data results in poorer performance on original samples while enhancing robustness.

Many researchers have investigated face recognition solutions tailored for surveillance videos. In pursuit of achieving stable video surveillance, Baomansi et al. designed a review process based on the RPCA-PCP method [27], comparing it with the BMC dataset and showcasing the performance of 13 state-of-the-art RPCA methods. Zhang et al. proposed a framework for recognizing personnel in video surveillance scenarios by leveraging heterogeneous contextual information and facial features to address face recognition issues with low-quality data [28]. Mandal et al. introduced a robust visual analysis system for detecting driver fatigue in buses, which involves detecting the driver’s state based on head–shoulder detection, face detection, and eye detection [29]. Liu et al. presented a PRO framework based on deep neural networks [30], which not only utilizes multimodal data from large-scale video surveillance, such as visual features and camera locations, but also constructs its own dataset of surveillance videos to ensure accuracy. Ding et al. proposed a trunk–branch ensemble CNN to enhance the robustness of CNN features against pose variations and occlusions [31]. This model extracts complementary information from the entire face image and patches cropped around facial components, achieving state-of-the-art performance on three popular video face databases. Wang et al. introduced a deep learning-based method for face recognition in real-world surveillance videos [32]. Through face detection, tracking, and labeling, they automatically and incrementally constructed a new dataset of target real-world surveillance videos and then fine-tuned a convolutional neural network with the labeled dataset. Mahdi designed a system for real-time monitoring using cameras [33], which consists of two steps: face detection using the Viola–Jones method and face recognition using the Kanade–Lucas–Tomasi algorithm as a feature tracker and PCA for identifying specific individuals. In 2018, Deng et al. chose an AdaBoost-based face detection algorithm to detect faces [34] and implemented a face recognition algorithm based on LBPHFace to create a laboratory management system using face recognition. Jose et al. implemented an intelligent multi-camera face recognition surveillance system using FaceNet and MTCNN algorithms on Jetson TX2 [35]. The proposed portable system tracks objects or suspects using camera IDs/locations and timestamps and records their status in a database through multiple camera installations. Wang et al. used an improved MTCNN algorithm for face detection [36], optimizing MTCNN and replacing the network feature extraction module in FaceNet with MobileNet for face recognition. They also designed a face recognition-based laboratory access control system. In 2023, Dong et al. adopted the DRN algorithm for super-resolution of low-resolution images and then performed face recognition using ArcFace [37], designing a smart classroom management system.

With the frequent use of university laboratories, an increasing number of issues have emerged. To better implement open management of laboratories, it is necessary to identify individuals entering and exiting the laboratories. Traditional face recognition methods rely heavily on high-definition facial images as input, and when faced with low-resolution faces, recognition may fail due to insufficient features. While increasing the clarity of camera equipment can address the issue of low resolution, it is limited by high costs and the substantial maintenance expenses associated with such equipment. Another approach is to reconstruct images using super-resolution techniques to convert low-resolution facial images into high-resolution ones, but as resolution decreases, facial features gradually diminish, leading to poorer reconstruction results. Furthermore, issues such as occlusion can arise due to factors like camera placement and personnel movement. Therefore, a better recognition solution is needed for facial recognition in surveillance scenarios.

This paper proposes a low-resolution face recognition method for application in laboratory surveillance scenarios. In this work, the backbone feature extraction network is improved, and the latest face recognition techniques and multi-object tracking algorithms are integrated into the network to address face recognition under practical surveillance conditions with low resolution.

Currently, there are several main factors that hinder face recognition in laboratory surveillance: the complex classroom environment often results in excessively small face resolutions, making detection and recognition difficult. Additionally, there are issues of misrecognition caused by occlusion and pose changes due to personnel movement. To address these problems, the research is divided into two parts:

1. Small Face Detection: In this paper, the WiderFace dataset is used to divide the training and testing sets. An improved face detection algorithm based on Retinaface is employed to complete the face detection task in laboratory surveillance. The improvement mainly involves incorporating SPD-Conv into the original Retinaface. Compared to traditional convolutions, this method maintains high performance while having fewer computations and achieving higher perception, thereby enhancing its performance in small target detection.
2. Small Face Recognition: In response to the decline in detection accuracy for low-resolution faces observed in current face recognition algorithms such as FaceNet, AdaFace is selected as the face recognition algorithm. During the face recognition process, misrecognition may occur due to occlusion caused by personnel movement. To address this, the ByteTrack multi-object tracking algorithm is integrated into AdaFace. Kalman filtering and the Hungarian algorithm are used to track the IDs of already recognized individuals, preventing the need for re-recognition. Finally, comparative experiments demonstrate that the improved method outperforms existing common face recognition algorithms.

## 2. Materials and Methods

Face recognition is one of the important research topics in the field of computer vision. It consists of face location, face alignment, and face classification [38]. Specifically, the first step is to detect faces and locate their positions in the image. Then, the preprocessed and cropped main face region is input into the backend network for face feature extraction and face matching. The main issues with current face recognition are not only the loss of features due to low resolution but also the scarcity of low-resolution datasets and the difficulty in applying them to practical applications.

### 2.1. Small-Sized Face Detection

RetinaFace is the latest single-stage face detection model proposed by Insight Face in 2019. This model is based on the structure of RetinaNet, utilizing deformable convolutions and a dense regression loss [8]. This paper leverages RetinaFace, which traditionally employs two types of backbone feature extraction networks: ResNet and MobileNet. Among them, ResNet outputs three effective feature layers {C3, C4, C5} from the convolutional blocks conv3_x, conv4_x, and conv5_x. This detection network is pre-trained and initialized using the ImageNet dataset. It adopts a Feature Pyramid Network (FPN) to extract features with rich semantic information using a top-down pyramid and lateral connections. Learning from the successful designs of Single Shot Headless Face Detector (SSH) [39] and PyramidBox [40], it employs separate context modules following the FPN to expand the receptive fields of pre-detection regions and enhance reasoning capabilities, thereby efficiently computing the corresponding multi-task losses.

This paper proposes to improve RetinaFace using Space-to-Depth Convolution. RetinaFace’s performance tends to decline rapidly when faced with tasks involving low-resolution images or small objects. This is due to the inevitable loss of fine-grained information and the learning of ineffective features when using strided convolutions or pooling layers.

This paper reconstructs RetinaFace using TensorFlow and experimentally validates the selection of ResNet as the backbone network for implementing the face detection model. Although ResNet’s powerful feature extraction capabilities have been widely applied in face detection, its performance tends to decline rapidly when faced with tasks involving low-resolution images or small objects due to the inevitable loss of fine-grained information and the learning of ineffective features caused by strided convolutions and pooling layers.

As the number of convolutional and pooling layers increases, it does not necessarily lead to better learning outcomes; instead, issues such as gradient vanishing, gradient exploding, and degradation arise. The prediction performance tends to worsen as the number of layers deepens. ResNet proposes a method where, when building a deep network by stacking new layers onto a shallow network, the added layers can be made to learn nothing and merely replicate the features of the shallow network. In this way, the new layers become identity mappings, ensuring that the performance of the deep network is consistent with that of the shallow network, thereby addressing the degradation problem. Compared to traditional convolutional networks, ResNet introduces shortcut connections that are connected to the input of the second activation function. In ResNet, this operation where the output equals the input is referred to as an identity mapping, which is the key to the residual structure.

**Figure 1:**
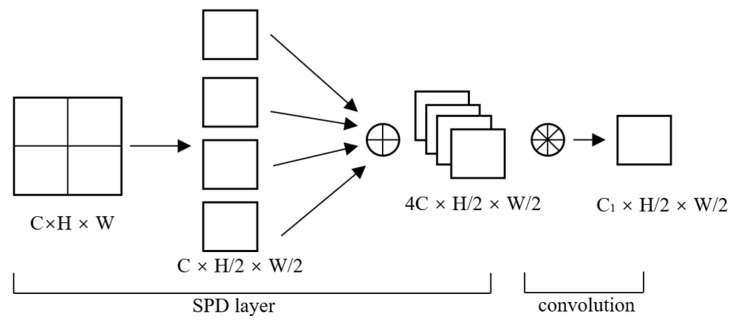
For an input feature map with dimensions C × H × W, downsampling is first performed. Assuming a scale factor of 2, the original C×H×W feature map is divided into four sub-feature maps of dimensions C × H/2 × W/2 each. This stage increases the depth of the feature map by reducing its spatial resolution. Next, these sub-feature maps are concatenated to form a new feature map with dimensions 4C × H/2 × W/2. This stage preserves the information from the original feature map while reducing its spatial resolution. Finally, a convolutional layer with a stride of 1 is applied to the new feature map. At this point, every pixel in the feature map is covered by the convolutional kernel, ensuring no information is skipped. The result is a new feature map with dimensions C_1_ × H/2 × W/2.

SPD-Conv [41] is a novel convolutional module whose primary purpose is to enhance performance when dealing with low-resolution images and small-sized objects. As shown in Figure 1, SPD-Conv adopts a new approach by utilizing an SPD layer combined with a non-strided convolutional layer to address this issue. It processes the original feature map through a series of transformations, resulting in a decrease in spatial resolution and an increase in the number of channels. Subsequently, a non-strided convolutional layer is applied to obtain more discriminative feature representations.

**Figure 2:**
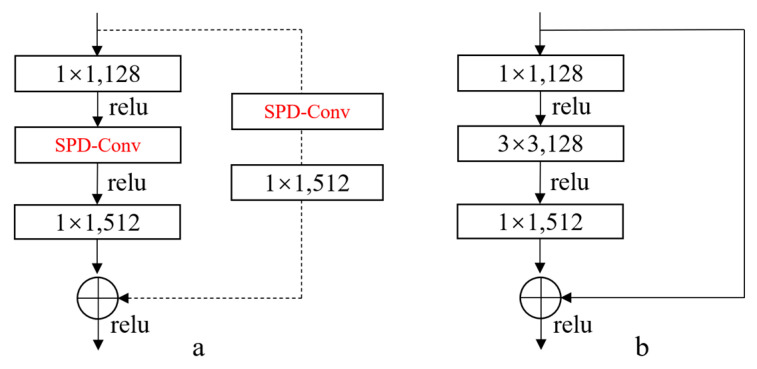
Take conv3_4 in ResNet50 as an example. The first residual block in each of the series of residual structures corresponding to conv3_x, conv4_x, and conv5_x are dotted-line residual blocks. This is because the first layer of these series of residual structures has the task of adjusting the shape of the input feature map. Figure (**b**) shows that the original Resnet three-layer residual element is first reduced by a 1 × 1 convolution, then by 3 × 3 convolution, and finally by 1 × 1 by ascending dimension. In addition, if the input and output dimensions are different, you can do a linear mapping transformation dimension for the input, and then connect the layers behind it. As shown in (**a**), after the first dotted-line residual block, solid-line residual blocks are connected, and the convolutional layer with a stride of 2 in the first dotted-line residual block is replaced with SPD-Conv.

To address the issue of information loss as the network depth increases when dealing with low-resolution images, we propose an improvement to the residual structure of ResNet, as shown in Figure 2. We select ResNet as the backbone feature extraction network and replace the convolutional layers with a stride of 2 with SPD-Conv. This modification helps prevent the loss of important information for low-resolution images and small-sized objects due to downsampling.

**Figure 3:**
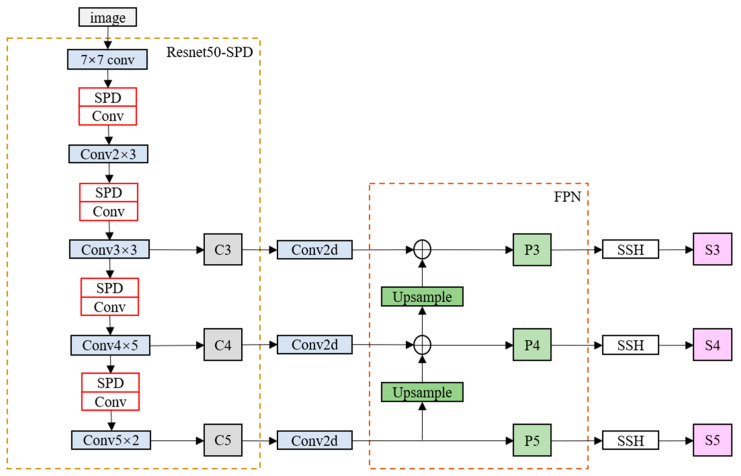
The improved network structure diagram of RetinaFace has the red portions representing the modified convolutional blocks from Figure 2. After obtaining the three effective feature layers S3, S4, and S5, classification prediction, bounding box regression for faces, and facial landmark detection are performed. Subsequently, non-maximum suppression (NMS) is applied to filter out the bounding box with the highest score within each region.

**Figure 4:**
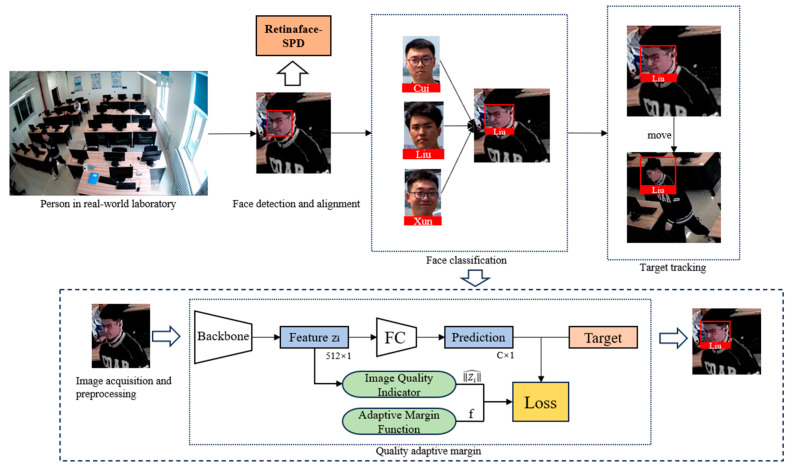
The process of the proposed face recognition method.

After modifying the backbone feature extraction network, we obtain three feature maps with different shapes, as shown in Figure 3. RetinaFace constructs an FPN (Feature Pyramid Network) structure using these three effective feature layers. Firstly, 1 × 1 convolutions are used to adjust the number of channels in these three feature layers. Then, upsampling and addition (Add) operations are performed for feature fusion. Finally, three feature layers are obtained, and the SSH (Single Shot Head) module is used to enhance the receptive field. After obtaining these three effective feature layers, prediction results are obtained through them. The face detection part corresponds to the RetinaFace-SPD section in Figure 4. Finally, based on the characteristics of surveillance scenarios with low-resolution images, we reconstruct RetinaFace using TensorFlow to accelerate the training speed.

### 2.2. Quality-Adaptive Margin for Face Recognition and Motion Occlusion Issues

Face classification differs from general object classification due to the challenging distinction between intra-class and inter-class feature variations in practical applications. The biggest challenge in large-scale face classification is optimizing the loss function to enhance intra-class compactness and inter-class separability for highly similar faces [42]. The Additive Angular Margin Loss (ArcFace) aims to enhance the discriminative power of learned deep features, thereby maximizing the separability of face classes [9]. However, the detection process heavily relies on clear face images and scenarios with minimal noise, leading to poor performance in identity recognition in surveillance scenarios. Due to the blurring and degradation of face images, face recognition in low-quality images and videos can result in the loss of relevant identity information. AdaFace [21] proposes an image quality-adaptive loss function that assigns different weights to samples of varying difficulty based on image quality. It adapts the margin function based on the phenomenon where the angular margin scales with training difficulty, optimizing hard samples when image quality is high and ignoring extremely hard samples when image quality is low. Moreover, it does not require additional modules to compute image quality but directly uses the correlation between feature norms and image quality, which is greater than the correlation between probability outputs and image quality.

AdaFace adjusts its function adaptively based on image quality indicators, as illustrated in the face classification section of Figure 4. When image quality is low, it does not emphasize hard samples, whereas when image quality is high, it emphasizes hard samples. By utilizing a margin-based loss function, the learned features are made sufficiently discriminative. The model can automatically assess the quality of images and differentiate between high-quality and low-quality images during the recognition process by assigning higher gradient scales to high-norm features far from the decision boundary and higher gradient scales to low-norm features close to the decision boundary.

**Figure 5:**
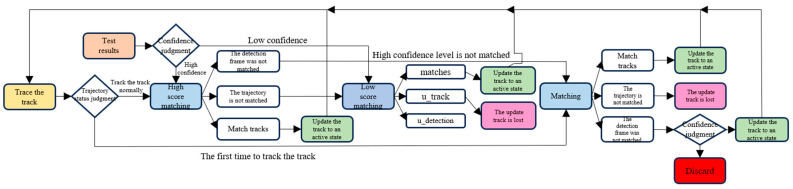
The ByteTrack flowchart represents the ByteTrack class, which handles processes such as trajectory creation, updating, and deletion. It features a primary method called update that continuously updates trajectory paths through the integration of predicted bounding boxes and existing calculations. The working principle of tracking involves processing each frame individually while also considering the context of consecutive frames. After distinguishing high-score and low-score bounding boxes, different treatments are applied based on the results. Finally, unmatched high-score detection boxes are reassessed, and if they meet the criteria, they are designated as new trajectories.

For scenarios with multiple individuals in laboratory surveillance footage and low resolution, issues such as re-identification and recognition failure can arise when the movement of individuals obscures the recognition targets. To address these issues, the ByteTrack multi-object tracking algorithm is incorporated into the existing AdaFace face recognition system. The ByteTrack algorithm inputs a video sequence, a detector, and a detection threshold. As shown in Figure 5, the algorithm outputs the trajectories of the video, with each frame containing the bounding boxes and IDs of the objects. For each frame in the video, the detector (Det) is first used to predict the bounding boxes and prediction scores. Then, based on the detection score threshold, the bounding boxes are classified into two categories: Det(high) and Det(low). After separating the bounding boxes, a Kalman filter is used to predict the new positions in the current frame for each trajectory T. By calculating the Intersection over Union (IOU) between the detected bounding boxes and the predicted bounding boxes, the Hungarian algorithm is finally used to match the IOU and return successful and failed trajectories. Instead of directly discarding low-score bounding boxes that may result from occlusion, the algorithm performs a secondary matching for these low-score bounding boxes, optimizing the issue of ID switching caused by occlusion during the tracking process. This avoids the need for secondary matching of already matched faces due to factors such as occlusion.

When dealing with severely occluded and overlapping trajectories, a series of strategies are employed: 1. The detection boxes in ByteTrack are classified based on confidence levels, dividing them into high-confidence and low-confidence groups. High-confidence detection boxes are used for initial matching, while low-confidence detection boxes are utilized in subsequent matching. Low-confidence detection boxes that have not been deleted continue to participate in the evaluation of subsequent frames, which helps maintain tracking even when the target is occluded. When a target is severely occluded or overlapped, the confidence of its detection box may decrease. However, ByteTrack helps to restore the identity of occluded or overlapped targets by retaining low-confidence detection boxes and reassessing their status in subsequent frames. 2. ByteTrack assigns a life cycle to each trajectory. If a trajectory does not match any detection box within a certain period, it will be deleted, avoiding fragmented trajectories caused by false detections or missed detections. For detection boxes that do not match any trajectory but have a sufficiently high confidence level, a new tracking trajectory will be created, which helps resume tracking after the target reappears from occlusion. 3. ByteTrack uses a Kalman filter to predict the movement of tracked objects, enabling the prediction of the target’s position in the next frame to assist in tracking even when the target is occluded or even disappears. 4. ByteTrack minimizes the use of ReID models. Instead of relying on identity matching, ByteTrack relies more on the positional overlap and motion continuity between detection boxes and trajectories for matching.

This paper proposes a multi-face recognition method tailored for laboratory surveillance scenarios. As shown in Figure 4, images of individuals captured from a self-constructed laboratory setting are input into the designed face recognition system for automatic processing. The obtained images are fed into an improved backbone feature extraction network for feature extraction. Face detection is achieved through the Feature Pyramid Network (FPN) and Single Shot MultiBox Detector (SSH) detection network, which outlines the faces. AdaFace is utilized to classify and match the detected faces with those recorded in the database. Finally, multi-object tracking is employed to track the classified faces, preventing issues such as misrecognition and re-identification caused by occlusion and movements of individuals.

## 3. Results

### 3.1. Dataset

**Figure 6:**
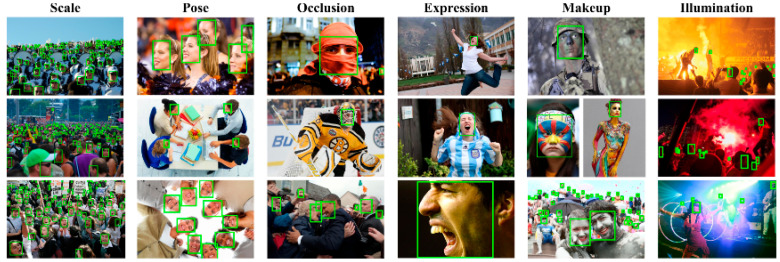
The Widerface dataset contains images categorized by various factors, including blur (degree of blurriness), expression (facial expression), illumination (lighting conditions), occlusion (degree of obstruction), and pose (facial orientation). Furthermore, it is divided into a total of 62 different categories based on different scenarios.

Given the complexity of laboratory personnel scenarios, the WiderFace dataset is more suitable for this experiment. The WiderFace dataset was first released in 2015, containing 32,203 annotated images with a total of 393,703 face data points. This is shown in Figure 6, each face is annotated with detailed information. We divided the WiderFace dataset into a training set and a test set and used it to train RetinaFace in order to verify the effectiveness of the reconstructed RetinaFace model.

### 3.2. Self-Built Laboratory Surveillance Dataset

**Figure 7:**
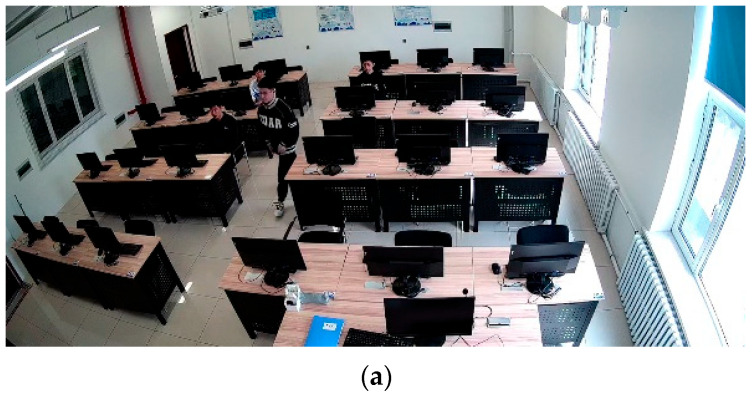

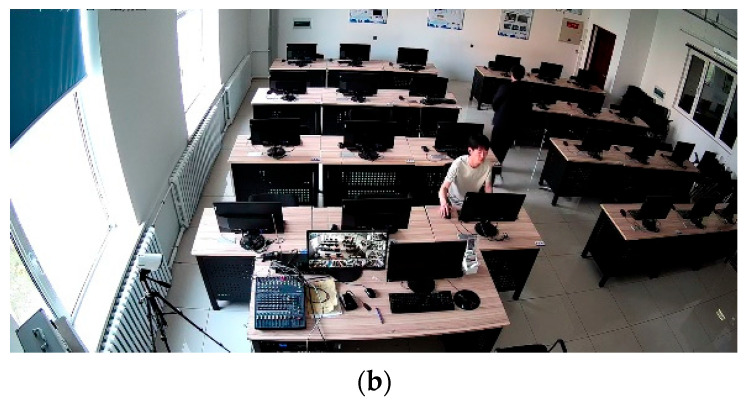
Figures (**a**,**b**) are the self-constructed laboratory surveillance camera student dataset simulates the daily use environment of the laboratory through different students entering and exiting the laboratory. The camera’s position is located at the top-left corner, and the resolution is 1080p (1920 × 1080).

**Figure 8:**
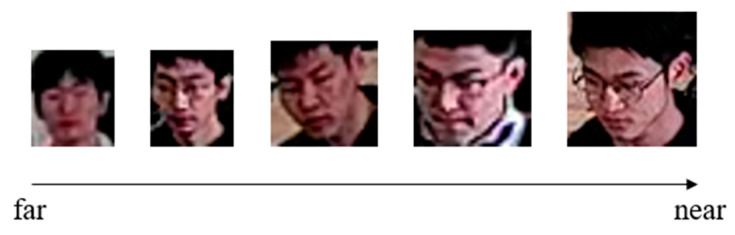
The size of faces captured at different distances in real-world scenarios.

The laboratory environment in this study is sourced from surveillance footage captured within computer rooms in university laboratories, as shown in Figure 7. The footage originates from surveillance cameras positioned above and to the left rear of the students in actual laboratory settings, with a resolution of 1920 × 1080. A total of 5344 valid images were extracted. By simulating various scenarios that may occur in daily laboratory use among students within the university, we recreated issues such as low facial resolution due to different seating distances and occlusion during movement. To test the feasibility of our method under extreme conditions, we also included recognition scenarios with high illumination, nighttime infrared camera footage, low light conditions, and recognition from greater distances. As illustrated in Figure 8, this figure demonstrates the size of extracted low-resolution faces. The pixel size of the farthest test subject is 25 × 30, with the smallest face size under extreme conditions being only 16 × 19 pixels. The closest test subject has a pixel size of 51 × 53.

### 3.3. Experimental Environment Setup

The experimental setup is as follows: the images used for training are of size 640 × 640; the Batch Size is set to 16; the maximum learning rate is 10-3, and the minimum learning rate is 10-5; and the model is trained for a total of 30 epochs to observe the decreasing rate of the loss function. The GPU used is RTX 1050 Ti, the Python version is 3.7, the PyTorch version is 1.8.0, the optimizer is SGD, and the processor is i5-8300H.

### 3.4. Experimental Evaluation Metrics

The experiment is mainly divided into two parts: the first part focuses on face detection, and the second part focuses on face recognition. During the face detection phase, this paper verifies the effectiveness of face detection using the test set divided from the Widerface dataset. To objectively evaluate the effectiveness, recall, precision, and mean Average Precision (mAP) are selected as evaluation metrics. Recall refers to the proportion of true samples that are correctly identified by the algorithm among all true samples. N_TP_ represents the number of samples correctly predicted by the network, while N_FN_ represents the number of samples falsely predicted by the network.

(1) $$\;V_{recall}=\frac{N_{TP}}{N_{TP}+N_{FN}}$$

Precision represents the proportion of samples that are determined to be true by the system among those that are already confirmed as true samples. The meanings of N_TP_ and N_FN_ are the same as those used in the calculation of recall:

(2) $$V_{precision}=\frac{N_{TP}}{N_{TP}+N_{FP}}$$

N_TP_ stands for the number of true samples that the network correctly predicts as “true”. N_FP_ represents the number of false samples that the network incorrectly predicts as “true”. The mean Average Precision value is calculated by averaging the average precision across all categories for all images:

(3) $$V_{mAP}=\int_{0}^{1}V_{precision}\left(r\right)dr$$

During the face recognition phase, the self-built dataset of laboratory personnel is used for testing. The main evaluation metric is the accuracy rate of face recognition, denoted as TAR. Here, N_TR_ represents the number of correct recognitions, and N_AR_ represents the total number of test attempts:

(4) $$TAR=\frac{N_{TR}}{N_{AR}}\times 100\%$$

### 3.5. Face Detection Experiment

We employed MobileNet and ResNet as the backbone feature extraction networks for RetinaFace and conducted comparisons using common face detection models on the WiderFace test set. The results verified that ResNet, as the backbone feature extraction network, outperforms MobileNetV1. We set three levels of difficulty for different images, easy, medium, and hard, and calculated the mAP values at these three difficulty levels to observe the performance of different models:

Fast R-CNN and DSFD exhibit higher accuracy in face detection, as shown in Table 1. However, both are two-stage detection algorithms, which means despite their very high accuracy, they have slower detection speeds due to their large number of parameters and computational requirements. In contrast, the one-stage algorithm RetinaFace offers advantages in both speed and detection accuracy. When comparing different backbone feature extraction networks, MobileNet demonstrates lower detection accuracy than ResNet, with ResNet maintaining good performance across samples of three different difficulty levels.

**Figure 9:**
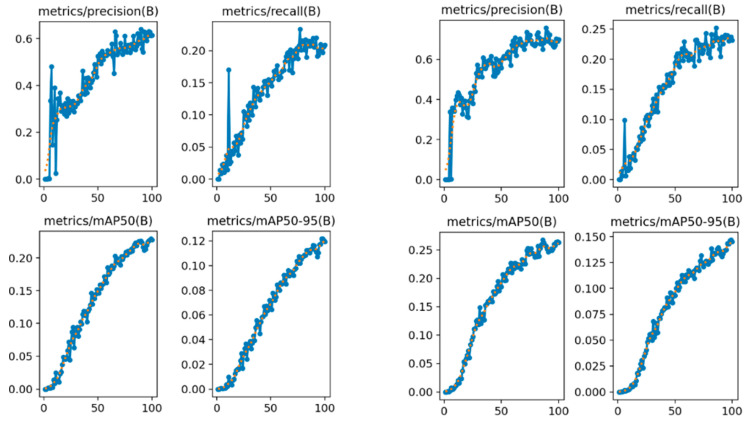
Comparison of precision, recall, and mAP before and after improvement using SPD-Conv.

The comparison of precision, recall, and mean Average Precision (mAP) before and after the improvement of SPD-Conv is shown in Figure 9. The four groups on the left represent the results before the improvement, while the four groups on the right represent the results after the improvement. Before the improvement, the Retinaface model exhibited significant fluctuations in precision and recall values as the threshold changed. The overall precision was below 0.6, and the overall recall was below 0.2. After the improvement, the fluctuations in precision and recall of the model were smaller, and there was a noticeable overall improvement. The overall precision was significantly above 0.6, and the recall could reach 0.25. This demonstrates that the Retinaface model improved with SPD-Conv is more capable of performing face detection in low-resolution scenarios.

**Figure 10:**
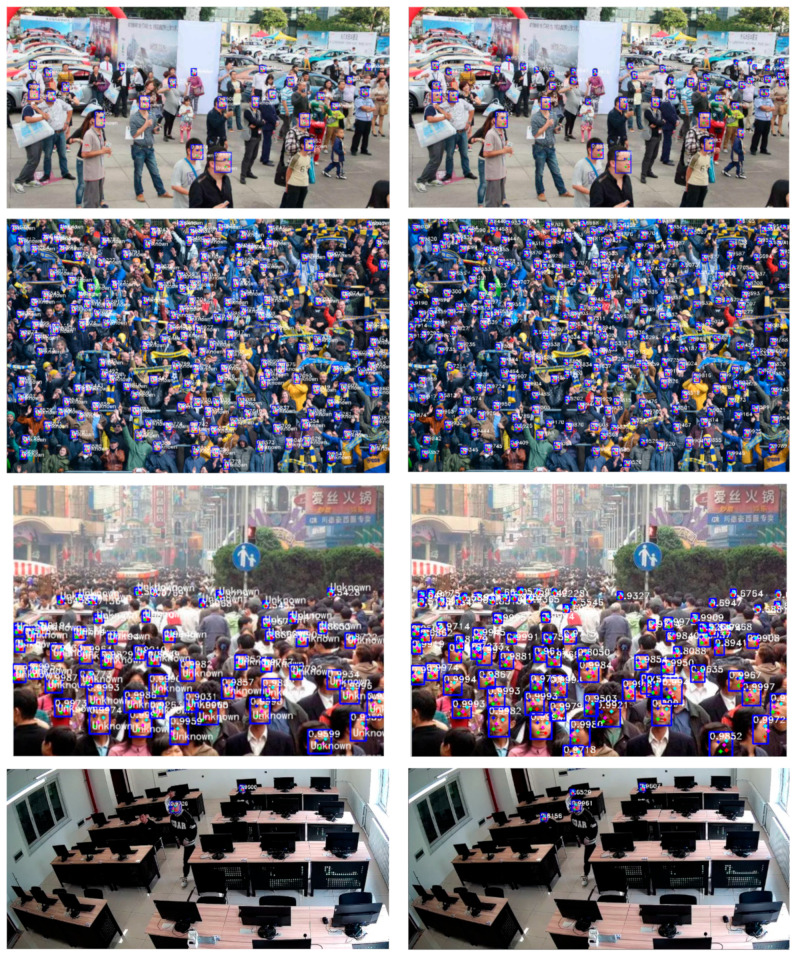
Comparison of face detection images using MobileNetV1 versus using ResNet50 as the backbone feature extraction network. The left side uses MobileNetV1, and the right side uses Resnet50. Obviously, it works better with Resnet50.

A comparison between the original RetinaFace and the improved version is presented below. As shown in Table 2, the improvements not only accelerated the training speed but also enhanced the accuracy of face detection. We set 30 epochs to compare the training speeds and the face recognition accuracy of the trained models. The reconstructed RetinaFace exhibited faster training speeds. In this paper, images featuring different numbers of people, varying distances, and complex scenes were used to test the accuracy of face detection. As shown in Table 3, the recognition accuracy is compared before and after the improvement in three different experimental environments. A comparison of the face detection accuracy between the two versions is shown in Figure 10. For complex and densely packed small faces, the sample results intuitively demonstrate that using ResNet as the backbone feature extraction network significantly outperforms MobileNet.

**Figure 11:**
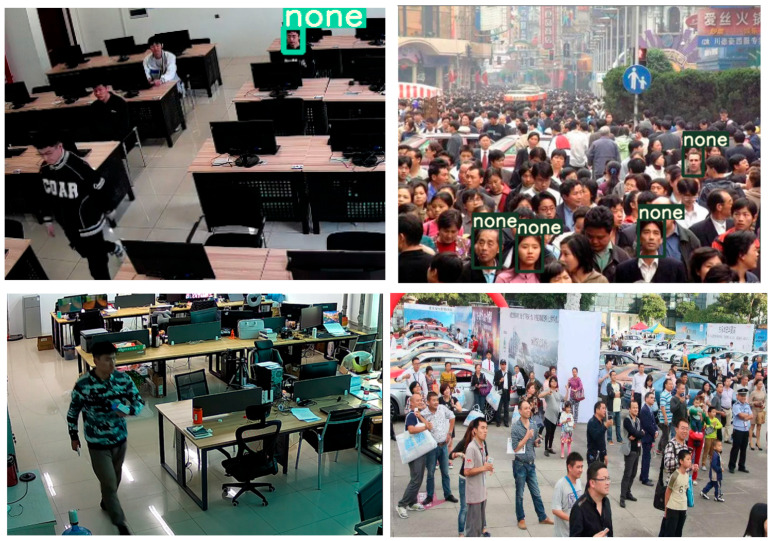
Face detection results using YOLO object detection algorithm.

RetinaFace combines deep learning and multi-task learning strategies to achieve high precision and efficiency in face detection. As shown in Figure 11, the face detection performance of YOLOv8-face is illustrated. In scenarios with extremely dense faces and low resolution, there are a large number of missed detections or even detection failures. This is because object detection algorithms such as YOLO prioritize real-time performance and generalizability. When encountering low-resolution face images, the detection accuracy is compromised, leading to missed detections at extreme resolutions. Additionally, due to the limited availability of face detection datasets currently, and the fact that YOLO algorithms require a large number of training images, a small dataset can also adversely affect detection performance.

On the Widerface dataset, 3226 annotated face images were selected as the test set. With the environment remaining constant, a series of comparative experiments were conducted using popular deep learning-based object detection algorithms (such as Faster-RCNN, RetinaFace, YOLO, etc.) to verify the feasibility of the proposed face detection scheme. The results, as shown in Table 4, indicate that our method achieves significant improvements in both accuracy and recall compared to other methods.

### 3.6. Face Recognition Experiment

**Figure 12:**
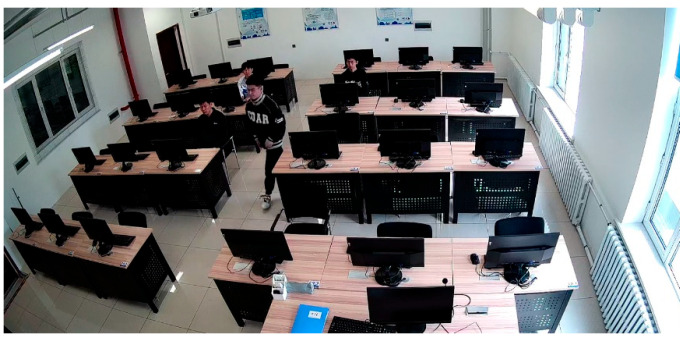
The image shown is an original image from a self-built laboratory monitoring scenario, where face recognition has not been performed.

The face recognition experimental data in this paper are sourced from two parts: 1. A self-built laboratory surveillance dataset. 2. The heterogeneous face recognition benchmark dataset IJB-S. As shown in Figure 12, to verify the feasibility of our method, we conducted comparative experiments with various common face recognition algorithms. In practical detection scenarios, there are not only issues of low face resolution but also problems such as cluttered scenes caused by furniture and equipment, as well as occlusion due to people’s movements.

When conducting detection on the IJB-S dataset, as shown in Table 5, the accuracy of face recognition is clearly demonstrated. When using Adaface, the recognition accuracy significantly outperforms all baselines, with improvements in the average performance across all four ranks. This indicates that Adaface will perform better in real-world application scenarios such as laboratory surveillance. As shown in Table 6, our method also exhibits excellent performance in our self-built dataset. We selected the most common super-resolution method in low-resolution application scenarios for comparison, and our method demonstrated superior performance both before and after personnel movement.

**Figure 13:**
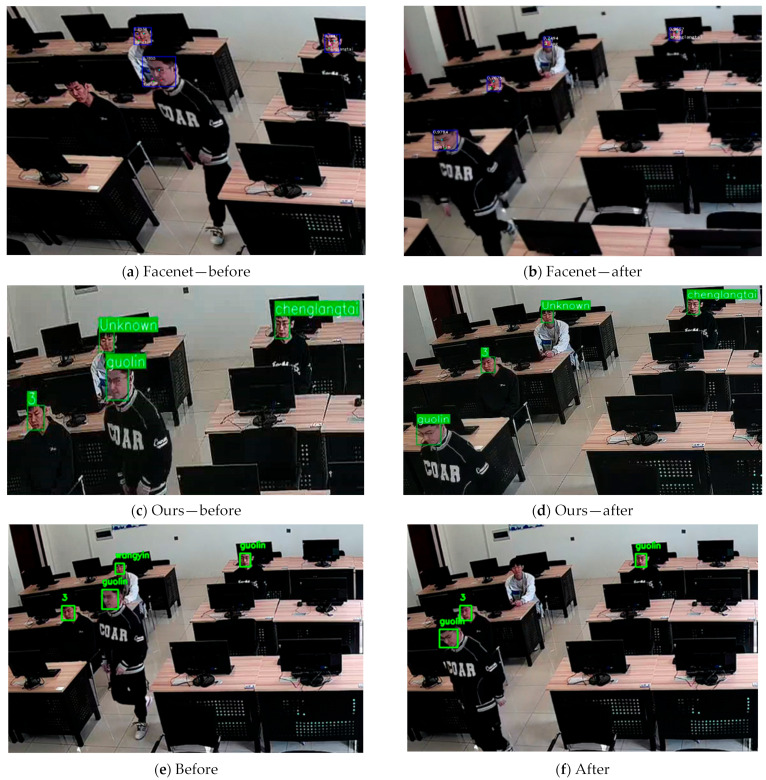
Face recognition using super-resolution reconstruction methods and face recognition using AdaFace classification, with the last group representing the case where no tracking is performed.

We incorporated a super-resolution reconstruction algorithm into Facenet for self-built laboratory surveillance comparison. Figure 13a,c demonstrate that despite an improvement in recognition accuracy after applying super-resolution reconstruction, there are still cases of missed detections and identity matching errors. When individuals move, their features change accordingly due to changes in position, which leads to variations in the effectiveness of super-resolution reconstruction and subsequently causes identity matching errors in recognition. For instance, in Figure 13a,b, after the person moves, an identity matching error occurs, resulting in a false match of a previously successfully matched face. In Figure 13c,d, after the person moves, due to the incorporation of a multi-object tracking algorithm, re-identification of already matched faces is not performed, and continuous tracking is maintained along the movement trajectory. The results indicate that, in a laboratory surveillance camera environment, both traditional recognition methods and super-resolution reconstruction methods struggle with identity matching. The method proposed in this paper improves the recognition accuracy for face recognition in laboratory surveillance scenarios and is more suitable for environments where personnel frequently move, such as laboratories.

**Figure 14:**
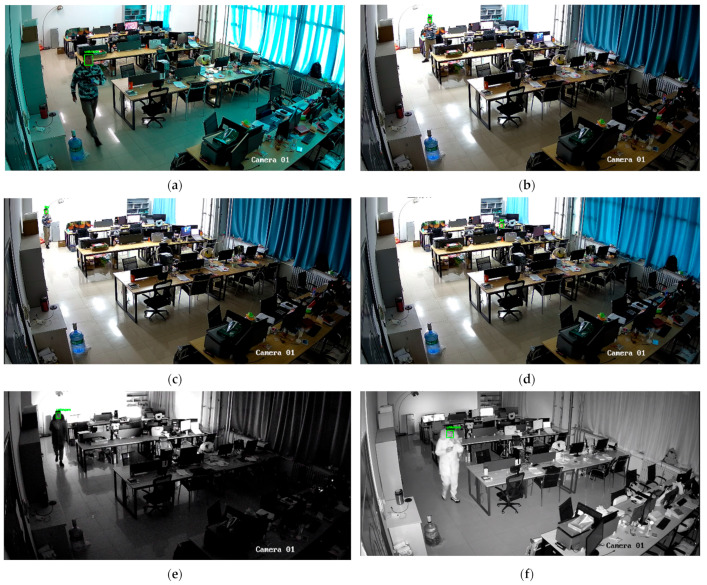
Experimental results in various special scenarios under real laboratory surveillance conditions. Figure (**a**) is the blur caused by the movement of the person, resulting in recognition failure, Figures (**b**–**d**), for a smaller size of the face, can still be recognized successfully, Figures (**e**,**f**) is infrared surveillance shooting, recognition failure.

In a larger laboratory setting, surveillance cameras positioned farther away were selected, and testers were asked to move quickly to test the performance under motion blur conditions. During the day, in the evening, and at night with lights off, face recognition effectiveness was assessed using infrared surveillance cameras. As shown in Figure 14, despite the distance from the surveillance cameras, the recognition results remained accurate. However, when the testers moved quickly, causing motion blur, due to the extremely low resolution, a significant amount of detail was lost, leading to recognition failures. During the daytime and under adequate lighting conditions, recognition was accurate. But when infrared cameras were used, recognition also failed due to the loss of facial features.

### 3.7. Ablation Experiment

To evaluate the contributions of adding the ByteTrack tracking module and the improved network based on SPD-Conv to face detection capability, experiments were conducted using real-life laboratory scenarios as the benchmark for assessment. The experimental results are shown in Table 7, with the last row representing the method proposed in this paper. Through comparative experiments, it was found that although face recognition accuracy can be improved without incorporating the tracking algorithm, the emergence of situations such as occlusion due to the movement of individuals can lead to a significant drop in accuracy during secondary identity recognition. When using CAface as the face detection algorithm, the performance is superior to adaface, but CAface is pre-trained on the backbone of adaface, so the improvement in accuracy is not significant. Moreover, adaface achieves better results when combined with the ByteTrack method.

## 4. Discussion

This paper investigates the issues associated with the application of traditional face recognition in practical scenarios of open university laboratories and computer rooms. It summarizes the development of face recognition algorithms in the direction of deep learning and the advancements in low-resolution face recognition. Furthermore, a method for low-resolution face recognition in laboratory scenarios based on RetinaFace and AdaFace is proposed. ResNet50-SPD is utilized as the backbone network for face detection and the RetinaFace face detection model is reconstructed. This method demonstrates excellent performance in detecting small-sized faces. For low-resolution face recognition, the Quality-Adaptive Margin approach stands out in terms of performance. Compared to classical techniques, our method exhibits superior performance and effectiveness in scenarios with low resolution, cluttered backgrounds, and frequent personnel movements, such as those found in surveillance systems in university laboratories and computer rooms. In a self-constructed environment, the proposed method achieved exceptional recognition performance. However, in cases of no lighting, a significant loss of effective information led to recognition failure. Similarly, rapid personnel movement resulted in the loss of facial details, causing recognition failure.

This work has only achieved offline face detection and recognition. However, in practical laboratory applications, not only is accuracy emphasized, but also real-time performance. In subsequent work, we will attempt to implement our method on embedded devices to perform real-time face recognition by capturing images. Additionally, laboratory management involves not only personnel management but also equipment management. Future work will continue to investigate and research these aspects of equipment management.

## Figures and Tables

**Table 1:** Comparison of mAP performance for face detection between several common face detection algorithms and the algorithm we used.

Model	Easy AP	Medium AP	Hard AP
Fast-Rcnn	96.38	94.92	89.92
MTCNN	85.10	82.36	60.84
DSFD	94.61	93.72	88.02
Yolov8-face	92.34	86.35	81.34
Retinaface mobilev1	89.77	86.98	74.75
Retinaface resnet50	94.76	93.22	84.92

**Table 2:** Comparison of convergence speed of the training loss function between the original RetinaFace and the improved RetinaFace.

Epoch	Retinaface_Loss	Retinaface-SPD_Loss
1	2.2149	1.7170
5	1.4833	1.1937
10	1.0883	0.8474
20	0.9124	0.5199
30	0.8401	0.3787

**Table 3:** Comparison of face detection accuracy between the original RetinaFace and the improved RetinaFace in different scenarios and under varying numbers of people.

Experiment	Retinaface	Retinaface-SPD
1	47.5%	96.1%
2	62.2%	90.3%
3	58.5%	92.5%

**Table 4:** Comparison of precision, recall, and mAP between several common face detection algorithms and the algorithm we used.

Model	Precision	Recall	AP
HighDimLBP	30.95	50.79	34.51
WebFace	45.22	82.84	45.49
Softmax	76.53	83.48	75.23
YOLOv8-face	91.03	82.62	86.53
Ours	96.12	90.42	91.46

**Table 5:** Comparison of the IJB-S dataset.

Dataset	Model	Rank-1	Rank-5
MS1MV2 [9]	ArcFace	56.39	62.43
	PFE	50.84	60.79
	URL	61.48	65.23
	ArcFace + aroFace	61.64	67.60
	Ours	65.26	70.53
WebFace4M [43]	ArcFace	68.29	72.43
	ArcFace + aroFace	70.94	75.54
	Ours	70.43	76.29

**Table 6:** Comparison of face recognition accuracy on a self-built laboratory surveillance dataset.

Model	Before Moving	After Moving
Facenet + DRN	75.41%	53.62%
Yolov8 + arcface	55.94%	43.36%
Ours	84.36%	83.79%

**Table 7:** Ablation experiment results (bold indicates the method proposed in this paper).

Face Detection	Face Recognition	Tracking	Before Moving	After Moving
Retinaface-SPD	Facenet	ByteTrack	30.32%	29.24%
	CAface	ByteTrack	85.46%	81.89%
	Adaface	None	86.47%	79.69%
	Adaface	ByteTrack	84.36%	83.79%
Retinaface	AdaFace	ByteTrack	81.75%	80.46%
	Adaface	None	81.45%	77.38%

## Data Availability

The raw data supporting the conclusions of this article will be made available by the author upon request.
